# Prevalence of *GCKR* rs1260326 Variant in Subjects with Obesity Associated NAFLD and T2DM: A Case-Control Study in South Punjab, Pakistan

**DOI:** 10.1155/2023/6661858

**Published:** 2023-10-04

**Authors:** Tayyaba Nisar, Kashan Arshad, Zahid Abbas, Maira Ali Khan, Sohail Safdar, Rehan Sadiq Shaikh, Ali Saeed

**Affiliations:** ^1^Institute of Molecular Biology and Biotechnology, Bahauddin Zakariya University, Multan 60800, Pakistan; ^2^Department of Pediatric Endocrinology and Diabetes, Pediatric Unit-1, Allied Hospital, Faisalabad 38800, Pakistan; ^3^PHRC, Nishtar Hospital, Multan 60000, Pakistan; ^4^Centre for Applied Molecular Biology, University of Punjab, Lahore, Pakistan; ^5^Department of Pediatric Oncology and Medical Microbiology, University Medical Center Groningen, University of Groningen, Groningen 9713, Netherlands

## Abstract

The glucokinase regulatory protein (GCKR) regulates glycogen metabolism and insulin secretion, and the *GCKR* rs1260326 is a putative single nucleotide polymorphism (SNP) associated with metabolic disorders including nonalcoholic fatty liver disease (NAFLD) and type 2 diabetes mellitus (T2DM). This study was conducted to investigate the genetic association of the *GCKR* rs1260326 in NAFLD and T2DM in our population. NAFLD (*n* = 103), T2DM (*n* = 100), and control (*n* = 100) samples were collected and genotyped for *GCKR* rs1260326 by tetra-arm PCR. The genetic variant *GCKR* rs1260326 was significantly linked with NAFLD and T2DM, while the *GCKR* rs1260326 was significantly associated with the progression of obesity only in NAFLD subjects. The frequency of the C allele (mutant) was higher in both NAFLD (*f* = 0.69) and T2DM (*f* = 0.66) subjects as compared to healthy controls of NAFLD (0.52) and T2DM (*f* = 0.32). The frequency of the C allele was also positively linked with the progression of obesity in both diseases. The frequency of the C allele was 0.66, 0.67, and 0.74 in NAFLD normal weight, overweight, and obese subjects, respectively, while the frequency of the C allele was 0.60, 0.60, and 0.74 in T2DM in normal weight, overweight, and obese subjects, respectively. Homozygous mutant (CC) was 53% in both NAFLD and T2DM subjects, while heterozygous mutant (CT) was 15.53% in NAFLD and 22% in T2DM subjects. Wild-type allele (TT) was 31.06% in NAFLD and 25% in T2DM subjects. In conclusion, the *GCKR* rs1260326 is a highly prevalent SNP in NAFLD and T2DM subjects, which possibly contributed to obesity, insulin resistance, and metabolic disorders in our population.

## 1. Introduction

Glucokinase regulatory protein, encoded by *GCKR,* is an inhibitory protein of glucokinase (GCK) and is expressed in the liver and *β*-cells of Islets. GCK is a glucose phosphorylating enzyme which is important in glucose-stimulated insulin release [1]. The *GCKR* is a highly pleiotropic gene on chromosome 2p23.3 with 19 exons encoding 625 amino acids. Therefore, the GCKR gene plays a pivotal role in various metabolic and biochemical pathways. Thus, the rare exonic mutations in *GCKR* are associated with several metabolic dysfunctions including elevated triglycerides and glucose levels [2]. GCKR inhibits GCK by forming an inactive heterodimer in an allosteric manner concerning glucose levels [3]. The activity of GCKR is increased by fructose-6-phosphate and antagonized by fructose-1-phosphate [4]. GCKR is a vital candidate protein related to glycogen metabolism and insulin secretion. However, genetic variations in this gene can lead to irregular blood glucose levels and decreased glucose responsiveness to develop insulin resistance, a condition called type 2 diabetes mellitus (T2DM). T2DM is a multifactorial metabolic disorder characterized by high plasma glucose levels resulting in insulin resistance or impaired *β*-cell function [5, 6]. The *GCKR* rs1260326 genetic variant is functionally relevant comprising of an amino acid substitution as C to T coding for a proline-to-leucine at position 446 (P446L) (Supplementary Figure 1) [7]. A missense variant in the *GCKR* rs1260326 is associated with abnormal fasting glucose levels and a higher risk of T2DM development [8]. Similarly, genome-wide association studies (GWASs) also reported that functional variants of the *GCKR* including rs1260326 are associated with low plasma insulin and fasting plasma glucose, as well as higher fasting and postprandial serum triglyceride levels [9, 10].

GCKR-mediated GCK inhibition maintains normal glucose and insulin levels, which play a pivotal role in hepatic *de novo* lipogenesis (DNL), a metabolic pathway that involves in the synthesis of fatty acids from carbohydrates when in excess. However, genetic polymorphism in the *GCKR* rs1260326 is associated with impaired hepatic lipid metabolism and secretion of very low density lipoprotein (VLDL), which can lead to hyperlipidemia and hepatic fat accumulation, and is significantly associated with the development of nonalcoholic fatty liver disease (NAFLD) in obese children and adolescents [7]. Therefore, an impaired cross-talk of GCKR and GCK along with elevated insulin and glucose levels promotes the DNL and subsequent hepatic steatosis, which leads to another metabolic condition called NAFLD [11]. NAFLD is a complex disease that results from multiple factors including environmental factors (western or high-fat diet), obesity, epigenetic factors, and genetic factors including polymorphism in disease-susceptible genes. The genetic factors associated with NAFLD and its underlying mechanisms are not fully understood. The diet-induced obesity is considered a primary event in the onset and development of NAFLD [11]. However, many studies also reported the role of single nucleotide polymorphism within the *GCKR* gene, in the development of NAFLD even independent of a high-fat diet, BMI, or the age of the subjects [12–15]. Furthermore, a previous study also reported a significant association of the *GCKR* rs1260326 with high fasting glycemia in the young Mexican population, without a significant association with obesity [16]. Thus, multiple previous studies have reported a significant genetic association between *GCKR* rs1260326 and NAFLD with and without obesity [12–15]. Previously, NAFLD was considered a part of metabolic syndrome (MS) and associated with obesity and T2DM [17]. Meta-analysis and genome-wide association studies (GWASs) have also shown a significant association of the *GCKR* rs1260326 with NAFLD in many populations worldwide including Iran [18], Swedish [19], Japanese [20], Danish [21], Pakistan [22], and China [23]. In Pakistan, prevalence of T2DM and NAFLD is 17.1% and 14%, respectively, while the prevalence of NAFLD in subjects with T2DM is 32–72% [24]. In Punjab, Pakistan, prevalence of T2DM in males and females is 12.14% and 9.83%, respectively [25]. Meta-analysis and GWAS analysis indicate that the *GCKR* rs1260326 has possibly impaired the hepatic lipid metabolism and significantly contributes to the development of obesity and obesity-associated metabolic disorder including NAFLD and T2DM. Thus, this study was conducted to evaluate the genetic association and contribution of *GCKR* rs1260326 to induce obesity in NAFLD and T2DM subjects in a local population of South Punjab.

## 2. Materials and Methods

### 2.1. Study Subject and Ethical Approval

In this study, a total of three hundred and three (*n* = 303) subjects were included (cases = 203 and controls = 100). The blood samples were collected from nonalcoholic fatty liver disease (NAFLD) (*n* = 103) and type 2 diabetes mellitus (T2DM) subjects (*n* = 100). Age- and sex-matched healthy control (HC) subjects (*n* = 100) were also collected for both diseases (*n* = 50 each). All samples were collected from Nishtar Hospital, Multan, Pakistan, under the signed Memorandum of Understanding (MoU) between Bahauddin Zakariya University and Nishtar Medical University, Multan, after ethical approval by the Institutional Review Board (IRB) of BZU (case number IMBB-334b). Informed written consent was also obtained from all the participants of the study as per the Declaration of Helsinki from all enrolled subjects. Samples were collected and transported to the molecular pathology laboratory of the Institute of Molecular Biology and Biotechnology, B.Z University, Multan, and stored at 4°C for further analysis.

Inclusion criteria for the NAFLD group were based on the confirmation of fatty liver duly confirmed with ultrasound irrespective to age, sex, and BMI. All fatty liver subjects suffering from hepatic cirrhosis, hepatocellular carcinoma, anaemia (bone marrow transplant), and alcohol consumption were excluded from this group. All T2DM subjects included in this study were adults (age >35) without insulin dependency, while subjects suffering from type 1 diabetes, gestational diabetes, maturity-onset diabetes, anaemia, chronic kidney disease, cardiovascular diseases, and alcohol consumption were excluded from this group. The subjects with a high-fat dietary intake were also excluded from both studies.

### 2.2. Physical and Biochemical Analysis for Sample Preparation

The demographic data included age, marital status, gender, weight, height, smoking, alcohol consumption family history, systolic blood pressure (BP), and diastolic blood pressure (BP). Body mass index (BMI) and fat content (FC) parameters were calculated using BMI calculator as previously described [18] and presented in Table 1. The biochemical parameters related to NAFLD including ALT (alanine aminotransferase), AST (aspartate aminotransferase), total bilirubin, and alkaline phosphatase were analyzed in disease and control subjects. Similarly, FBG (fasting blood glucose), RBG (random blood glucose), and Hb1Ac (haemoglobin A1c) were analyzed in T2DM subjects and related control subjects as previously described [26].

### 2.3. The *GCKR* rs1260326 Genetic Variant and Molecular Detection

The *GCKR* rs1260326 variant was selected according to the basic genotypic and disease association information available in South Punjab, Pakistan. The basic information of the genetic variant *GCKR* rs1260326 is presented in Supplementary Table 1. Furthermore, DNA was isolated from fresh blood samples by using the phenol-chloroform method [27, 28]. Tetra-arms PCR (Bio-Rad T100 Thermal Cycler, Hercules-California, USA) was used to detect this variant by using DNA (1 ng), master mix (Vazyme Biotech co., Nanjing, China) (7.5 *μ*L), and 1 *μ*L each primer (5 *μ*M). A total of 40 cycles were performed with initial denaturation at 95°C for 5 min, denaturation at 94°C for 45 sec, annealing at 55°C for 1 min, extension at 72°C for 35 sec, and final extension at 72°C for 10 min.

The primers were designed by Primer 1 online tool for tetra-arms PCR for the *GCKR* rs1260326 as previously described [29]. The following primers were used for the detection of single nucleotide polymorphism (SNP): outer forward: 5′–GTGGTCTTCATTTTCACCCTG–3′, inner forward: 5′–CCGTGGGTCAGACCTTTCT–3′, outer reverse 5′–CTGAGCCCCTTACTGCAGAT–3′, and inner reverse: 5′–ACGGCTGGACTCTCAACG–3′. The homozygous wild type allele (TT) amplified 240 bp, while the homozygous mutant allele (CC) amplified 338 bp. The heterozygous (CT) amplified both amplicons (240 bp and 338 bp). However, the outer primer set amplified 551 bp in all samples as a PCR internal quality control.

### 2.4. Statistical Analysis

The chi-square test was performed using IBM SPSS Statistics V.23 software (IBM, Chicago, USA) for statistical significance between case (NAFLD and T2DM) and control subjects. The Hardy–Weinberg equilibrium was used to compare the genotype and allelic frequencies. Data were presented as mean ± SD or the number of cases. Binary logistic regression was also performed along with the Hosmer–Lemeshow goodness-of-fit test to find associations. *P* values (*P* ≤ ^*∗*^, ≤^*∗∗*^, and ≤^*∗∗∗*^) were considered significant.

## 3. Results

**Figure 1:**
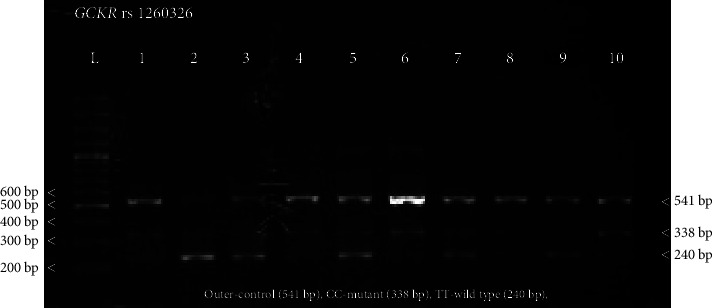
Tetra-arms PCR and gel electrophoresis of *GCKR* rs1260326 variant. Tetra-arms PCR was optimized and analyzed by gel electrophoresis. DNA was extracted from all samples and amplified with *GCKR* rs1260326 specific primers. This figure shows 1 to 10 representative samples from NAFLD subjects. The 541 bp amplicon was considered enteral control for all PCR reactions. The 338 bp amplicon was identified as a homozygous mutant C/C, while 240 bp was considered a homozygous wild type T/T. Tetra-arm primer amplified 338 bp in mutant variants and 240 bp in wild-variants and both amplicons in heterozygous variants. Amplicon length was identified with a 100 bp DNA ladder (L).

All samples were stratified and grouped into three categories including normal weight (BMI ≤25), overweight (BMI 25 to ≤30), and obese (BMI >30) to analyze the association of obesity with the *GCKR* (rs1260326) genetic variants in nonalcoholic fatty liver disease (NAFLD), type 2 diabetic mellitus (T2DM), and control subjects. Genotyping of the *GCKR* rs1260326 variant was performed. Homozygous mutant (CC) amplified 338 bp and homozygous wild type (TT) amplified 240 bp, while heterozygous (CT) amplified both 338 bp and 240 bp amplicons (Figure 1).

### 3.1. Demographic Data of NAFLD and T2DM Subjects

Demographic and clinical parameters have been analyzed for all subjects and presented (Tables 1 and 2). Age, disease history, hypertension, duration, BMI, fat content, and blood pressure were significantly associated with NAFLD. Gender, family history, exercise, BMI, fat content, FBGL, RBGL, and Hb1Ac were also significantly associated with T2DM. Furthermore, the *GCKR* rs1260326 genetic variant was also significantly associated with the development of NAFLD and T2DM. Most of the other risk factors were commonly associated with both disease subjects except gender, age, disease history, smoking, blood pressure, and RBGL (Supplementary Table 2).

### 3.2. Genotypic and Allelic Frequency of the *GCKR* rs1260326 in NAFLD Subjects

The distribution of *GCKR* rs1260326 genotype has been shown for case-control groups in NAFLD subjects (Table 3). Allelic and genotypic frequencies of normal (BMI <25), overweight (BMI 25-to <30), and obese (BMI >30) NAFLD subjects were calculated and compared with the control subjects. The frequency of the C allele in obese NAFLD was 74.19%, while the C allele frequency was 52% in control subjects. The frequency of the T allele (wild type) was 25.81% in NAFLD obese subjects, whereas the T allele frequency was 48% in healthy controls. The allelic frequency of the mutant allele (CC) was higher in NAFLD subjects than that of the wild type allele (TT) (Table 3). Furthermore, the frequency of mutant C allele also increased with the progression of obesity in NAFLD subjects. The liver damage markers including ALT (61.20 ± 110.41) and AST (61.20 ± 110.41) were significantly elevated in NAFLD normal weight subjects, while ALT and AST were in the normal range in overweight and obese subjects. However, bilirubin and alkaline phosphatase levels were normal range in all NAFLD subjects (Supplementary Table 3).

### 3.3. Genotypic and Allelic Frequency of the *GCKR* rs1260326 in T2DM Subjects

The distribution of *GCKR* rs1260326 variant has been shown for case-control groups in T2DM subjects (Table 4). Allelic and genotypic frequencies of normal (BMI <25), overweight (BMI 25-to <30), and obese (BMI >30) T2DM subjects were calculated and compared with the control subjects. The frequency of the C allele (mutant type) in obese T2DM was 74.07%, whereas the T allele frequency was 32% in healthy controls. The frequency of the T allele (wild type) was 25.93% in obese T2DM subjects, whereas the T allele frequency was 68% in control subjects. The allelic frequency of the mutant allele (CC) was higher in T2DM subjects than that of wild type allele (TT). The frequency of the mutant C allele also increased with the progression of obesity in T2DM subjects.

### 3.4. Association of the *GCKR* rs1260326 with Obesity in NAFLD and T2DM

Allelic and genotypic frequencies of the mutant allele (CC) in all NAFLD and T2DM subjects were 68.93% and 65.5%, respectively, as compared to wild type allele (TT) in NAFLD (31.06%) and T2DM (34.5%) control subjects (Table 5). The genetic variant *GCKR* rs1260326 was significantly associated with both diseases (NAFLD and T2DM), and mutant type (CC) was the most prevalent genotype as compared to wild type (TT) in both diseases, especially in obese patients. Binary logistic regression has shown a significant association of the *GCKR* rs1260326 variant with obesity in NAFLD as compared to normal-weight subjects. However, all other combinations analysis did not show statistical significance (Table 6).

## 4. Discussion

The *GCKR* rs1260326, substitutes proline to leucine at position 446 of amino acid (P446L), is a genetic risk factor associated with obesity-associated nonalcoholic fatty liver (NAFLD) and type 2 diabetes mellitus (T2DM) in different population [9, 13, 30]. This study reports an association of the *GCKR* rs1260326 genetic variant with the development of NAFLD and T2DM in the obese subjects, in the local population of South Punjab, Pakistan. In this population, the mutant risk allele (C) of *GCKR* rs1260326 was highly prevalent in NAFLD (74.19%) and T2DM (74.07%) obese subjects as compared to wild type allele (T). While in some studies, the frequency of C allele was 42.6% in NAFLD [13] and T2DM subjects [15]. A genome-wide association study (GWAS) in Malaysia has reported a significant association of the *GCKR* rs1260326 with the development of hepatic steatosis, NAFLD, and nonalcoholic steatohepatitis (NASH) [31]. Single nucleotide polymorphism of the *GCKR* rs1260326 alters the adiponectin function and influences the lipogenesis pathway to increase susceptibility and severity of NAFLD and hepatic fibrosis [13]. In line with this, the mutant C risk allele of the *GCKR* rs1260326 has been reported genetic risk factor associated with NAFLD in obese subjects [30].

Obesity usually coexists with metabolic disorders including NAFLD and T2DM. Visceral obesity and high BMI were high risk factors, especially in both metabolic disorders. Previous studies indicated that the prevalence of obesity usually surpassed 90% of the subjects in NAFLD [8]. The development of obesity was also significantly linked with the *GCKR* rs1260326 mutant allele (C) especially in NAFLD subjects, while the frequency of mutant allele (C) was considerably higher in T2DM obese subjects but did not show a statistical significance in comparison to normal weight subjects. Thus, the *GCKR* rs1260326 was a genetic risk factor and significantly linked to NAFLD and T2DM in the local population, which can contribute to the development of obesity and insulin resistance. Similarly, the *GCKR* rs1260326 was also linked with hepatic fat metabolism and fat contents in T2DM [15].

Serval risk factors have also been linked to the development of obesity and NAFLD [32]. High-fat dietary uptake was considered a major contributory factor in the development of NAFLD. Similarly, this study also reports several other demographic risk factors associated with the development of NAFLD and T2DM without high-fat dietary uptake. In this study, the risk factors including age, disease history, diabetes, hypertension, duration, blood pressure, ALT, AST, and AP have shown a significant association with NAFLD development.

The association of *GCKR* rs1260326 has been repeatedly reported especially in NAFLD with a considerable contribution to liver damage and hepatic fibrosis [33]. The*GCKR* rs1260326 was associated with a higher chance of developing NAFLD and NASH with severe fibrosis [34] and increased hepatic *de novo* lipogenesis (DNL) in obese youngsters [35]. In this study, we also reported a significant elevation of liver damage markers (AST, ALT, and AP) in normal-weight NAFLD subjects; however, the liver damage markers were in the normal range with the progression of obesity in overweight and obese NAFLD subjects. Higher liver damage in normal-weight NAFLD subjects as compared to the overweight and obese NAFLD subjects was indicating a protective role of fat storage until overloading of fat in hepatocytes and inflammation. The persistent fat storage in hepatocyte possibly leads to loss of hepatocyte function and promotes the infiltration of inflammatory cells to contribute to hepatic steatohepatitis, NASH, and further liver damage [11]. A previous study indicated that proliferator-activated receptor *γ* (PPAR*γ*) mediated adipogenesis and browning of fat protected liver damage in NAFLD and improved insulin sensitivity [36]. This partially explained a possible mechanism of moderate liver damage in obese NAFLD subjects as compared to normal weight NAFLD subjects. However, circulatory markers also dropped with the progression of chronic liver disease, for example, AST and ALT levels could not rule out advance stages of chronic liver diseases [37]. This can be another explanation for low levels of liver damage markers with the advance stage of NAFLD in this study.

In this study, BMI, body fat, FBGL, RBGL, and Hb1Ac were significant risk factors associated with the development of T2DM. Similarly, the genetic polymorphisms in different genes such as CAMK2, IGF1, IRS1, GCKR, PPARG, GCK1, and KCTD1 are found associated with insulin resistance and T2DM [38, 39]. Similarly, genetic variations in the *GCKR* expression can also regulate glucose metabolism and insulin sensitivity in obesity-associated metabolic disorders including NAFLD and T2DM [5, 6]. The P446L substitution affects the ability of GCKR negative regulation of glucokinase in response to fructose-6-phosphate and increased the activation of hepatic glucose uptake, resulting in reduced circulating insulin and fasting glucose [40]. Other studies reported elevated insulin secretion and low plasma glucose levels associated with the *GCKR* rs1260326 in Danish diabetic populations [10, 41]. Similar to the previous studies, this study also reported a significant association of the *GCKR* rs1260326 in the mutant (CC) variants with T2DM subjects without a significant difference in HbA1c, fast, or random glucose levels as compared to the wild-type variants. However, obesity (body fat content and BMI) was considerably higher in the mutant-type T2DM subjects. Similar to previous studies [34], this study also indicated that the *GCKR* rs1260326 possibly contributed to the pathology of T2DM and insulin resistance through lipogenesis or obesity-associated pathways.

Similarly, the *GCKR* variant rs1260326 has been correlated with metabolic traits, such as higher levels of triglycerides and a higher incidence of dyslipidemia, without the onset of metabolic syndrome [3] and T2DM [9]. This indicated the possibility of two separate biological mechanisms which contributed to the development of metabolic disorders including metabolic syndrome, T2DM, and NAFLD.

## 5. Conclusion

The *GCKR* rs1260326 genetic variant contributed to the impaired hepatic lipid and glucose metabolism and promoted the development of metabolic disorders including NAFLD and T2DM. The *GCKR* rs1260326 mutant allele (CC) was considerably higher in subjects with obesity and insulin resistance independent of the high fat and glucose uptake. This study highlighted the importance of additional lipid metabolic pathways regulated by glucose metabolism and significantly contributed to the development of NAFLD and T2DM. This study indicated that the *GCKR* gene is also a novel therapeutic target in the treatment of obesity and obesity-associated metabolic disorders. The *GCKR* rs1260326 variant has potential research and clinical implications regarding to its impact on metabolism of glucose and lipid.

Future direction aspects of the *GCKR* rs1260326 include investigating the role in developing targeted therapies and personalized medicine as well as potential biomarker for the risk assessment of the disease. However, further studies are still required to better understand the underlying mechanisms and concluding the interaction with other environmental and genetic factors involved in lipid metabolism in glucokinase regulator (GCKR) deficient subjects.

## Figures and Tables

**Table 1:** The demographic data of NAFLD and control subjects.

Variables	NW (*n* = 34)	OW (*n* = 38)	OB (*n* = 31)	HS (*n* = 50)	*P* value
Gender (M/F)	17/17	14/24	6/25	21/29	0.467^NS^
Age (years, mean ± SD)	38 ± 11	41 ± 9	43 ± 9	27 ± 11	**<0.001** ^*∗∗∗*^
History of NAFLD (yes/no)	4/30	5/33	6/25	0/50	**0.004** ^*∗∗*^
Exercise (yes/no)	18/16	26/12	18/13	32/18	0.650^NS^
Smoking (yes/no)	3/31	2/36	2/29	0/50	0.059^NS^
Diabetes (yes/no)	9/25	15/23	12/19	0/50	**<0.001** ^*∗∗∗*^
Hypertension (yes/no)	4/30	10/28	9/22	0/50	**<0.001** ^*∗∗∗*^
NAFLD duration (weeks, mean ± SD)	44 ± 121	103 ± 183	95 ± 186	0.000 ± 0.000	**0.033** ^*∗*^
BMI (kg/m^2^, mean ± SD)	22.27 ± 1.73	27.15 ± 1.37	33.47 ± 3.14	20.82 ± 2.79	**<0.001** ^*∗∗∗*^
Fat content (%, mean ± SD)	25.20 ± 6.12	32.76 ± 5.97	42.64 ± 7.02	21.53 ± 5.23	**<0.001** ^*∗∗∗*^
Blood pressure (systolic/diastolic) (mmHg, mean ± SD)	120 ± 6/80 ± 5	123 ± 11/83 ± 9	122 ± 7/82 ± 7	120 ± 0/80 ± 0	**<0.001** ^*∗∗∗*^
*GCKR* (rs1260326) (CC/TT/CT)	18/7/9	19/6/13	18/3/10	9/7/34	**<0.001** ^*∗∗∗*^

NW: normal weight (BMI >18.5 to <25), OW: overweight (BMI 25 to<30), OB: obese (BMI >30), HS: healthy subjects, data are presented as mean ± SD, Chi-square test was used, and *P*  < 0.05^*∗*^, ≤ 0.01^*∗∗*^, and ≤ 0.001^*∗∗∗*^. Bold values indicate significantly associated risk factor with disease progression.

**Table 2:** The demographic data of T2DM and control subjects.

Variables	NW (*n* = 30)	OW (*n* = 27)	OB (*n* = 43)	HS (*n* = 50)	*P* value
Gender (M/F)	18/12	17/10	10/33	47/3	**<0.001** ^*∗∗∗*^
Age (years, mean ± SD)	52 ± 10	51 ± 9	51 ± 9	51 ± 10	0.588^NS^
Marital (yes/no)	26/4	25/2	40/3	43/7	0.350^NS^
Smoking (yes/no)	8/22	5/22	9/34	6/44	0.138^NS^
Family history (yes/no)	13/17	17/10	30/13	11/39	**<0.001** ^*∗∗∗*^
Exercise (yes/no)	19/11	12/15	16/27	40/10	**<0.001** ^*∗∗∗*^
Waist (In., mean ± SD)	38 ± 36	41.59 ± 3.09	45.72 ± 2.67	35.66 ± 3.08	**<0.001** ^*∗∗∗*^
BMI (kg/m^2^, mean ± SD)	22.24 ± 2.24	27.21 ± 1.35	33.44 ± 2.42	23.44 ± 3.35	**<0.001** ^*∗∗∗*^
Fat content (%, mean ± SD)	27.19 ± 6.78	32.72 ± 5.39	44.09 ± 5.03	24.25 ± 5.16	**<0.001** ^*∗∗∗*^
Blood pressure (systolic/diastolic) (mmHg, mean ± SD)	137 ± 24/88 ± 10	151 ± 35/94 ± 17	146 ± 43/94 ± 30	131 ± 27/87 ± 13	0.659^NS^/0.172^NS^
FBGL (mg/dl, mean ± SD)	191.78 ± 41.97	188.33 ± 35.45	196.44 ± 47.55	66.96 ± 9.87	**<0.001** ^*∗∗∗*^
RBGL (mg/dl, mean ± SD)	360.56 ± 72.92	248.14 ± 63.73	369.01 ± 71.51	124.54 ± 15.34	**<0.001** ^*∗∗∗*^
HbA1c (%, mean ± SD)	9.45 ± 1.38	9.23 ± 1.33	9.72 ± 1.62	3.92 ± 0.33	**<0.001** ^*∗∗∗*^
*GCKR* (rs1260326) (CC/TT/CT)	15/9/6	18/5/4	20/11/12	11/29/10	**<0.001** ^*∗∗∗*^

Data are presented as mean ± SD; FBGL (fasting blood glucose level); RBGL (random blood glucose level); HbA1c (haemoglobin A1C). Chi-square test was used and *P*  < 0.05^*∗*^, ≤ 0.01^*∗∗*^, and ≤ 0.001^*∗∗∗*^. Bold values indicate significantly associated risk factor with disease progression.

**Table 3:** Genotype and allelic frequencies of *GCKR* rs1260326 in obese NAFLD vs control.

Genotypes (*n* *=* total)	CC, *n*(%)	CT, *n*(%)	TT, *n*(%)	C allele frequency, *n*(%), *f*	T allele frequency, *n*(%), *f*
NW (*n* = 34)	18 (52.94%)	9 (26.47%)	7 (20.58%)	45 (66.18%), 0.66	23 (33.82%), 0.34
OW (*n* = 38)	19 (50%)	13 (34.21%)	6 (15.78%)	51 (67.11%), 0.67	25 (32.89%), 0.33
OB (*n* = 31)	18 (58.06%)	10 (32.25%)	3 (9.67%)	46 (74.19%), 0.74	16 (25.81%), 0.26
HS (*n* = 50)	9 (18%)	34 (68%)	7 (14%)	52 (52%), 0.52	48 (48%), 0.48

NW: normal weight (BMI >18.5 to <25), OW: overweight (BMI 25 to<30), OB: obese (BMI >30, CC: mutant type, CT: heterotype, TT: wild type. Allelic frequencies of genotypes were calculated according to Hardy–Weinberg equation.

**Table 4:** Genotype and allelic frequencies of *GCKR* rs1260326 in obese T2DM vs control.

Genotypes (*n* *=* total)	CC, *n*(%)	CT, *n*(%)	TT, *n*(%)	C allele frequency, *n*(%), *f*	T allele frequency, *n*(%), *f*
NW (*n* = 30)	15 (50%)	9 (30%)	6 (20%)	36 (60%), 0.60	24 (40%), 0.40
OW (*n* = 43)	20 (46.51%)	11 (25.58%)	12 (27.90%)	52 (60.47%), 0.60	34 (39.53%), 0.40
OB (*n* = 27)	18 (66.66%)	5 (18.51%)	4 (14.81%)	40 (74.07%), 0.74	14 (25.93%), 0.26
HS (*n* = 50)	11(22%)	29 (58%)	10 (20%)	68 (32%), 0.32	32 (68%), 0.68

NW: normal weight (BMI >18.5 to <25), OW: overweight (BMI 25 to <30), OB: obese (BMI >30, CC: mutant type, CT: heterotype, TT: wild type. Allelic frequencies of genotypes were calculated according to Hardy–Weinberg equation.

**Table 5:** Genotype and allelic frequencies of *GCKR* in NAFLD and T2DM subjects.

Genotypes (*n* *=* total)	CC, *n*(%)	CT, *n*(%)	TT, *n*(%)	C allele frequency, *n*(%), *f*	T allele frequency, *n*(%), *f*
NAFLD (*n* = 103)	55 (53.39%)	16 (15.53%)	32 (31.06%)	142 (68.93%), 0.6893	64 (31.06%), 0.3107
T2DM (*n* = 100)	53 (53%)	22 (22%)	25 (25%)	131 (65.5%), 0.655	69 (34.5%), 0.345

NW: normal weight (BMI >18.5 to <25), OW: overweight (BMI 25 to <30), OB: obese (BMI >30, CC: mutant type, CT: heterotype, TT: wild type. Allelic frequencies of genotypes were calculated according to Hardy–Weinberg equation.

**Table 6:** Association of *GCKR* rs1260326 with obesity in NAFLD and T2DM subjects.

Genotype	NW vs OW *P*value/OR (95% CI)	OW vs OB *P*value/OR (95% CI)	NW vs OB *P*value/OR (95% CI)
NAFLD	0.932/1.028 (0.546–1.933)	0.263/0.653 (1.333−1.282)	**0.024** ^*∗*^/2.641 (1.134–6.148)
T2DM	0.234/1.713 (0.706, 4.160)	0.603/0.811 (368, 1.786)	0.165/0.536 (0.223, 1.292)

NW: normal weight (BMI >18.5 to <25), OW: overweight (BMI 25-to <30), OB: obese (BMI >30). Binary logistic regression has been performed. *P*  < 0.05^*∗*^, ≤ 0.01^*∗∗*^, and ≤ 0.001^*∗∗∗*^. Bold values indicate significantly associated risk factor with disease progression.

## Data Availability

This is the first time these data are submitted for publication and hence these data are not publicly available.

## Supplementary Materials

Supplementary Materials Supplementary Table 1: the basic information regarding SNP selected in Southern Population, Multan, Pakistan. Supplementary Table 2: the risk factor comparison in both diseases (NAFLD and T2DM). Supplementary Table 3: association of obesity with liver damage in NAFLD subjects. Supplementary Figure 1: the diagram displaying the nucleotide sequence of the GCKR rs1260326 variant and proline to leucine substitution.
