# Supplementary material for: Prevalence of GCKR rs1260326 Variant in Subjects with Obesity Associated NAFLD and T2DM: A Case-Control Study in South Punjab, Pakistan
Source: J Obes. 2023 Oct 4;2023:6661858. doi: 10.1155/2023/6661858 (PMC10567336; doi:10.1155/2023/6661858)
Supplement: Supplementary Materials — Supplementary Table 1: the basic information regarding SNP selected in Southern Population, Multan, Pakistan. Supplementary Table 2: the risk factor comparison in both diseases (NAFLD and T2DM). Supplementary Table 3: association of obesity with liver damage in NAFLD subjects. Supplementary Figure 1: the diagram displaying the nucleotide sequence of the GCKR rs1260326 variant and proline to leucine substitution. [file 6661858.f1.docx]

#### **Prevalence of GCKR rs1260326 variants in obese subjects of NAFLD and T2DM: a case-control study in Southern Punjab, Pakistan**

Tayyaba Nisar^1^, Kashan Arshad^2^, Zahid Abbas^1^, Maira Ali Khan^1^, Sohail Safdar^3^, Rehan Sadiq Shaikh^1,4^, Ali Saeed^1,5*^

^1^Institute of Molecular Biology and Biotechnology, Bahauddin Zakariya University, Multan, Multan 60800, Pakistan.

^2^Department of Pediatric Endocrinology and Diabetes, Pediatric Unit-1, Allied Hospital, Faisalabad 38800 , Pakistan.

^3^PHRC, Nishtar Hospital, Multan 60000, Pakistan.

^4^Centre for Applied Molecular Biology, University of Punjab, Lahore, Pakistan.

^5^Department of Pediatric Oncology & Medical Microbiology, University Medical Center Groningen, University of Groningen, Groningen 9713 GZ, The Netherlands.

**Corresponding author**: Dr. Ali Saeed, Institute of Molecular Biology and Biotechnology, Bahauddin Zakariya University, Multan, Pakistan.

Email addresses: dralisaeed84@gmail.com, alisaeed@bzu.edu.pk, Tel.: +92-(0)-3226616269.

**Email addresses:** tayyabanisar247@gmail.com;kashan_arshad@hotmail.com; zahidabbas708060@gmail.com; mairaalikhan05@gmail.com, rehan.camb@pu.edu.pk, sohailpmrc@gmail.com, dralisaeed84@gmail.com

**SUPPLEMENTARY DATA**

**Supplementary table 1: The basic information regarding SNP selected in Southern Population, Multan, Pakistan**

| SNP | Variant type | MAF | Alleles |
| --- | --- | --- | --- |
| rs1260326 | Missense Variant | 0.29 | C>T |
| SNP: single nucleotide polymorphism; *GCKR*: Glucokinase Regulator; MAF: Minor allele frequency | | | |

**Supplementary table 2: The risk factor comparison in both diseases (NAFLD and T2DM) subjects**

| **Variables** | **NAFLD**  (n=103) | **T2DM**  (n=100) | ***P*-Value** |
| --- | --- | --- | --- |
| Gender  (M/F) | 37/66 | 55/45 | **0.006***** |
| Age  (Years, mean±SD) | 41±10 | 51±9 | **0.034*** |
| Family History  (Yes/No) | 15/88 | 60/40 | **<0.001***** |
| Exercise  (Yes/No) | 62/41 | 47/53 | 0.059 |
| Smoking  (Yes/No) | 7/96 | 22/78 | **0.002**** |
| BMI  (kg/m^2^, mean±SD) | 27.44±4.95 | 28.40±5.23 | 0.210 |
| Fat Content  (%, mean±SD) | 33.24±9.38 | 35.95±9.31 | 0.099 |
| Blood Pressure  (Systolic / Diastolic)  (mmHg, mean±SD) | 122±8/82±7 | 145±36/92±22 | **<0.001***** |
| RBGL  (mg/dl, mean±SD) | 139.03±93.89 | 360.84±69.76 | **<0.001***** |
| *GCKR* (rs1260326)  (CC/TT/CT) | 55/16/32 | 53/22/25 | 0.148 |
| **BMI**: (Body mass index); **RBGL**: Random blood glucose level. Data are presented in mean ± SD; *Chi*-Square test was used.& *p<0.05*, ≤0.01**, ≤0.001**** | | | |

**Supplementary table 3: Association of obesity with liver damage in NAFLD subjects**

| Variables | NW  (n=34) | OW  (n=38) | OB  (n=31) | *P-Value* |
| --- | --- | --- | --- | --- |
| AST  (U/L, mean±SD) | 61.20±110.41 | 41±27.05 | 43.12±35.47 | **<0.001***** |
| ALT  (U/L, mean±SD) | 54.47±50.90 | 53.02±49.81 | 44.74±47.96 | **<0.001***** |
| Bilirubin  (mg/dL, mean±SD) | 0.77±0.85 | 0.74±1.13 | 0.56±0.25 | 0.0816^NS^ |
| AP  (U/L, mean±SD) | 118.64±62.11 | 113±47.58 | 112.48±34.16 | **<0.001***** |
| ALT: alanine aminotransferase; AST: aspartate aminotransferase, AP: alkaline phosphatase. Data is presented in mean ± SD, *p<0.05*, ≤0.01**, ≤0.001**** | | | | |

**Supplementary figure 1: The diagram displaying the nucleotide sequence of the *GCKR* rs1260326 variant and proline to leucine substitution**

**
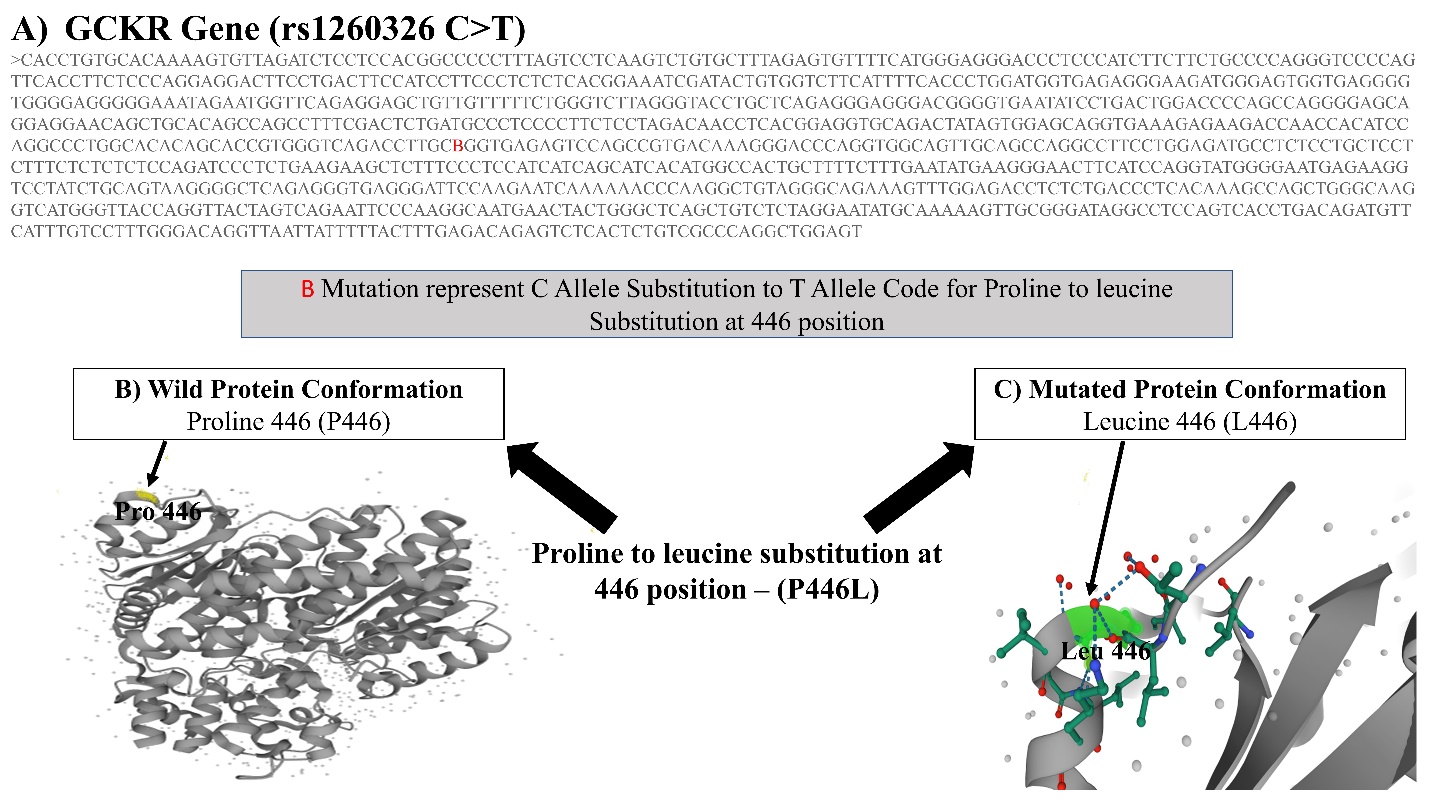
**
